# New Binary Locally Repairable Codes with Locality 2 and Uneven Availabilities for Hot Data

**DOI:** 10.3390/e20090636

**Published:** 2018-08-25

**Authors:** Kang-Seok Lee, Hosung Park, Jong-Seon No

**Affiliations:** 1 Samsung Electronics, Co. Ltd., Hwasung, Gyeonggi-do 18448, Korea; 2 School of Electronics and Computer Engineering, Chonnam National University, Gwangju 61186, Korea; 3 Department of Electrical and Computer Engineering, Institute of New Media and Communications, Seoul National University, Seoul 08826, Korea

**Keywords:** availability, distributed storage system (DSS), locality, locally repairable code (LRC)

## Abstract

In this paper, a new family of binary LRCs (BLRCs) with locality 2 and uneven availabilities for hot data is proposed, which has a high information symbol availability and low parity symbol availabilities for the local repair of distributed storage systems. The local repair of each information symbol for the proposed codes can be done not by accessing other information symbols but only by accessing parity symbols. The proposed BLRCs with k=4 achieve the optimality on the information length for their given code length, minimum Hamming distance, locality, and availability in terms of the well-known theoretical upper bound.

## 1. Introduction

Distributed storage systems (DSSs) which efficiently store information on several distributed nodes have been proposed [1,2]. The purpose of DSSs is to ensure reliable and efficient storage of information. The first techniques for DSSs were based on replication and have been adopted in various storage systems. However, the main disadvantage of replication is the large amount of storage overhead required, resulting in serious inefficiency as the amount of stored information increases. As a means of solving this problem, erasure coding schemes were proposed to achieve reliable storage of information with very small amounts of storage overhead compared to that required by replication methods [3].

However, it is well known that traditional erasure codes, such as Reed–Solomon (RS) codes, are not optimal for DSSs, because DSSs have different performance criteria. Specifically, these erasure codes do not have the optimal performance in local repair, which is one of the important criteria for DSSs. Local repair refers to a repair process that reconstructs the original data of an erasure symbol (node) using a small number of other symbols. During the local repair process, the repair bandwidth [4], disk input/output (I/O) [5,6,7], and locality [8] are known to be the main repair cost metrics. The repair bandwidth and disk I/O represent the number of bits communicated and read during the local repair process, respectively. In addition, locality refers to the number of symbols (nodes) participating in the local repair. Each of these metrics is considered in DSSs for different purposes, and their fundamental bounds for optimality have not been completely determined yet.

In particular, locality is considered to be important in applications of erasure codes in DSSs. In order to reduce the degree of locality, various studies have been done, and codes with a small locality are commonly referred to as locally repairable codes (LRCs) [8,9,10]. Recently, as another performance criterion associated with LRCs, availability was introduced [11,12]. Availability is defined as the number of disjoint sets of symbols which can be used to repair a symbol. In DSSs with LRCs, a high availability makes local repair flexible so that hot data are loaded without lagging.

In this paper, a new family of binary LRCs (BLRCs) is proposed to enhance performance of local repair in the DSSs. The proposed BLRCs have all-symbol locality two and uneven availabilities for the local repair of LRCs in DSSs. The proposed BLRCs also have a high information symbol availability and low parity symbol availabilities, which improve performance of the DSSs, especially for information data. In addition, most local repair groups of the proposed BLRCs have one information symbol and two parity symbols and thus we do not need to access other information symbols for the local repair of each information symbol. This property is desirable for information symbols in hot data storage systems. The proposed BLRC with k=4 can achieve optimality of the information length for a given code length and the minimum Hamming distance while maintaining the practically good locality and uneven availabilities.

The rest of the paper is organized as follows. In Section 2, for easy understanding of the conventional and the proposed LRCs, several notations, definitions, and fundamental properties are introduced. Subsequently, in Section 3, the new family of BLRCs with locality two and uneven availabilities is proposed and various characteristics of the proposed BLRCs are analyzed. Finally, conclusions are given in Section 4.

## 2. Preliminaries

### 2.1. Notations and Definitions

In this paper, all vectors and matrices are denoted with a boldface font. For vectors a and b of the same length, $\left[a\;b\right]$ and $\left[\begin{array}{c} a\\ b \end{array}\right]$ denote row-wise and column-wise concatenations of a and b, respectively. Similarly, for matrices A and B of the same column length, $\left[A\;B\right]$ denotes the row-wise concatenation of A and B. Also, for matrices C and D of the same row length, $\left[\begin{array}{c} C\\ D \end{array}\right]$ denotes the column-wise concatenation of C and D.

Suppose that a binary vector x has at least one zero element. For the given binary vector x, we define a random function $Z\left(x\right)$ to convert x to a binary vector of the same length by changing a randomly selected zero element to one. Let z(x) denote an instance of Z(x). For l≥1, we also introduce a random function $Z^{l}\left(x\right)$ as the composite function of $Z\left(x\right)$, which is recursively defined as

$$Z^{l}\left(x\right)=Z\left(z^{l-1}\left(x\right)\right).$$

where $z^{0}\left(x\right)$ is defined to be x. Then, the function $Z^{1}\left(\cdot \right)$ becomes equal to Z(·).

Let C be an $\left(n,k,d\right)$ linear code, which encodes *k* information symbols to a codeword of length *n*, that is, $c=\left(c_{0},c_{1},\cdots ,c_{n-1}\right)$ with a minimum Hamming distance of *d*. A k×n generator matrix G of code C is said to be in a systematic form if

$$G=\left[I_{k}\;P\right]$$

where $I_{k}$ denotes the identity matrix of size k×k corresponding to the systematic (information) part and P denotes a $k\times \left(n-k\right)$ matrix corresponding to the parity part. For the $\left(n,k,d\right)$ binary code C, if the k×n generator matrix G is in a systematic form, the $\left(n-k\right)\times k$ parity-check matrix H is easily obtained as

$$H=\left[P^{T}\;I_{n-k}\right]$$

where $P^{T}$ denotes the transpose of P.

### 2.2. Locally Repairable Codes

In this paper, an LRC of length *n*, information length *k*, locality *r*, availability *t*, and minimum Hamming distance *d* is referred to as an $\left(n,k,r,t,d\right)$ LRC. In an $\left(n,k,r,t,d\right)$ LRC, *n* symbols of any codeword have at least *t* disjoint groups, each of which includes at most *r* other symbols used to repair the erasure symbol. An LRC is sometimes denoted by $\left(n,k\right)$, $\left(n,k,r\right)$, or $\left(n,k,r,d\right)$ LRC depending on which parameters we deal with. If an $\left(n,k,r,t,d\right)$ LRC supports locality *r* for only *k* information symbols, it is referred to as an $\left(n,k,r,t,d\right)$ LRC with information symbol locality. On the other hands, if it supports locality *r* for all *n* symbols of codewords, it is referred to as an $\left(n,k,r,t,d\right)$ LRC with all-symbol locality. Similarly, $\left(n,k,r,t,d\right)$ LRCs can be classified based on the type of availability as follows.

**Definition 1** (Information-symbol availability)**.** *If an $\left(n,k,r,d\right)$ LRC supports availability t for local repair on each of k information symbols, it is referred to as an $\left(n,k,r,t,d\right)$ LRC with information symbol availability.*

**Definition 2** (All-symbol availability)**.** *If an $\left(n,k,r,d\right)$ LRC supports availability t for all local repair of each of n symbols, it is referred to as an $\left(n,k,r,t,d\right)$ LRC with all-symbol availability.*

Note that Definitions 1 and 2 are valid for a case in which an $\left(n,k,r,t,d\right)$ LRC achieves all-symbol locality.

Further, in order to consider a case that each symbol has different availabilities, a new definition for the availability is needed as follows.

**Definition 3** (All-symbol availability profile)**.** *For an $\left(n,k,r,d\right)$ LRC C, the all-symbol availability profile of C is defined as a vector $t=\left[t_{1},t_{2},\cdots ,t_{n}\right]$ of length n, where $t_{i}$ (≥1) denotes the availability for local repair of the i-th symbol of a codeword in C. The LRC C is denoted by $\left(n,k,r,t,d\right)$ LRC.*

If an $\left(n,k,r,d\right)$ LRC C has an all-symbol availability profile t, whose elements are not all identical, it is said that C has uneven availabilities.

### 2.3. Bounds for Optimality of LRCs

In pioneering researches on the bound for the optimality of LRCs, it was shown that the minimum Hamming distance *d* of an $\left(n,k,r,d\right)$ LRC C should satisfy an upper bound in Reference [8],

(1) $$d\leq n-k+2-\left\lceil \frac{k}{r}\right\rceil ,$$

which is a modification of the Singleton bound, where ⌈·⌉ denotes the ceiling function. Various constructions of LRCs achieving the bound in Equation (1) have been proposed [12,13,14,15].

Subsequently, a bound for $\left(n,k,r,d\right)$ LRCs was introduced [16] to additionally take the symbol size *q* into account compared to the bound in Equation (1). This bound indicates that the information length *k* of an $\left(n,k,r,d\right)$ LRC C over $F_{q}$ has the following upper bound,

(2) $$k\leq \min_{t\in Z^{+}}\left\{tr+k_{\mathrm{opt}}^{\left(q\right)}\left(n-t\left(r+1\right),d\right)\right\}$$

where $k_{\mathrm{opt}}^{\left(q\right)}\left(n,d\right)$ denotes the largest possible code dimension of an *n*-length code for a given alphabet size *q* and a given minimum distance *d*. Note that Equation (2) represents the bound for the information length *k*, whereas Equation (1) indicates that for the minimum Hamming distance *d*. The explicit constructions of the family of BLRCs in earlier works [16,17] achieve the bound in Equation (2).

Recently, another bound for $\left(n,k,r,t,d\right)$ LRCs was introduced in Reference [12]. This bound also takes both locality *r* and availability *t* into account similar to the bound in Equation (2), but it does not consider the symbol size *q*. It is derived for a case in which each local repair group has only one local parity symbol, where the local parity symbol denotes the parity symbol used for local repair.

(3) $$d\leq n-k-\left\lceil \frac{kt}{r}\right\rceil +t+1$$

## 3. A New Family of BLRCs

In this section, a new family of BLRCs is proposed and their locality and availability are analyzed. The proposed BLRC with the information length k=4 is found to be optimal in terms of the bound in Equation (2) for the given code length, locality, availability, and minimum Hamming distance.

### 3.1. Construction of New BLRCs

In this subsection, a new family of high-rate BLRCs with locality two and uneven availabilities is proposed. The construction of the proposed BLRCs requires the following intermediate procedure. Firstly, a *k*-tuple binary column vector $z_{k}$ with Hamming weight one is generated, where the position of the nonzero element is random. Based on $z_{k}$, k×k square matrices $P_{k,l}$ for 1≤l≤k−1 are constructed one by one by increasing *l* as

$$P_{k,l}=\left[Z^{l}\left(z_{k}\right)\;Z_{\left(1\right)}^{l}\left(z_{k}\right)\;\cdots \;Z_{\left(k-1\right)}^{l}\left(z_{k}\right)\right]$$

where $Z^{l}\left(z_{k}\right)$ is obtained for given $z^{l-1}\left(z_{k}\right)$ by the order of construction and $Z_{\left(i\right)}^{l}\left(z_{k}\right)$ denotes the *i* circularly downward-cyclic-shifted vector of $Z^{l}\left(z_{k}\right)$. Next, a k×k(k−2) matrix $P_{k}$ for the parity part of the generator matrix is generated by concatenating the matrices $P_{k,1}$, $P_{k,2}$, ⋯, $P_{k,k-2}$ as

(4) $$P_{k}=\left[P_{k,1}\;P_{k,2},\;\cdots ,P_{k,k-2}\right].$$

The construction of the proposed BLRCs is based on the construction of the generator matrix as follows.

**Construction** **1:** Let $G_{\left(n,k\right)}$ denote the systematic generator matrix of the proposed $\left(n,k\right)$ BLRC C. Then, a k×n generator matrix $G_{\left(n,k\right)}$ in the systematic form is constructed as

(5) $$G_{\left(n,k\right)}=\left[I_{k}\;P_{k}\right].$$

Note that the generator matrix $G_{\left(n,k\right)}$ has size of $k\times k\left(k-1\right)$ and code rate of $R=1/\left(k-1\right)$.

**Figure 1:**
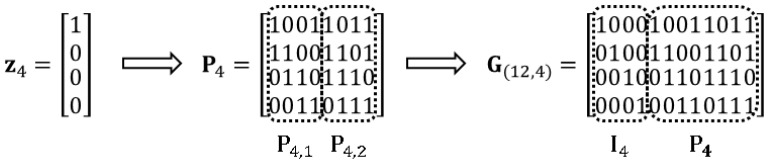
A generator matrix of the $\left(12,4\right)$ BLRCs in Construction 1.

**Example** **1.** *Figure 1 shows an example of the procedure of Construction 1 for the proposed $\left(n=12,k=4\right)$ BLRCs. For the generator matrix $G_{\left(12,4\right)}$, the column vector $z_{4}={\left(1\;0\;0\;0\right)}^{T}$ is used. The example BLRC has a code length of n=12 and a code rate of R=1/3.*

### 3.2. Locality and Availability of the Proposed BLRCs

In this subsection, the locality *r* and availability profile t of the proposed BLRCs are analyzed as the main performance criteria for local repair in LRCs. As noted above, in an $\left(n,k,r,t,d\right)$ LRC, the *i*-th symbol can be repaired using at least $t_{i}$ disjoint groups, each consisting of at most *r* other symbols. In case of an LRC with multiple locality values, there were some research results [18,19]. Our proposed BLRCs have a uniform all-symbol locality value but various values of availability. This is explained in the latter part of this subsection.

In general, the locality and availability of LRCs are easily analyzed from corresponding parity-check matrix as follows.

**Lemma** **1** ([17])**.** *An $\left(n,k\right)$ LRC has locality r if for every index i,1≤i≤n, its parity-check matrix has a row vector x, which has a Hamming weight at most r+1 and has a nonzero element in the i-th position.*

**Lemma** **2** ([12])**.** *An $\left(n,k,r\right)$ LRC has availability t if for every index i,1≤i≤n, there exist at least t row vectors, of which each commonly has a nonzero element in the i-th position and, disjointly, has other r nonzero elements in positions except i in its parity-check matrix.*

In the proposed BLRCs, all-symbols have locality two as follows.

**Theorem 1** (Locality of the proposed BLRCs)**.** *For an $\left(n,k\right)$ LRC according to Construction 1, all-symbol locality is r=2.*

**Proof.**  In the proposed BLRCs, for local repair, a parity-check matrix $H^{\prime }$ modified by elementary row operations is utilized instead of the original parity-check matrix H. The modification procedure for $H^{\prime }$ is as follows. The parity-check matrix in a systematic form is obtained from the generator matrix as

(6) $$H={\left[\begin{array}{c} \begin{array}{cccc} P_{k,1} & P_{k,2} & \cdots & P_{k,k-2} \end{array}\\ I_{n-k} \end{array}\right]}^{T}.$$

Let the parity-check matrix be represented in another form as

(7) $$H={\left[\begin{array}{cccc} Q_{k,1} & Q_{k,2} & \cdots & Q_{k,k-2} \end{array}\right]}^{T}$$

where the sub-matrices $Q_{k,l},1\leq l\leq k-2$, are n×k matrices. All of the row vectors of $Q_{k,l}^{T}$ have Hamming weight l+2, of which l+1 nonzero elements and one nonzero element are in their left sub-vector of length n−k and their right sub-vector of length *k*, respectively. Then, the parity-check matrix is modified as

(8) $$H^{\prime }={\left[\begin{array}{cccc} Q_{k,1} & Q_{k,1}+Q_{k,2} & \cdots & Q_{k,k-3}+Q_{k,k-2} \end{array}\right]}^{T}.$$

It is easily checked in Equation (6) that the row vectors in the identical positions of $Q_{k,l}^{T}$ and $Q_{k,l+1}^{T}$, 1≤l≤k−3, have Hamming distance three by construction, with one occurring in their left sub-vectors of length n−k and the other two occurring in their right sub-vectors of length *k*. Therefore, for $Q_{k,l}^{T}+Q_{k,l+1}^{T},\;1\leq l\leq k-3$, the Hamming weight of their row vectors is three. In addition, all of the column vectors in $Q_{k,l}^{T}+Q_{k,l+1}^{T},\;1\leq l\leq k-3$ have nonzero Hamming weights. According to Lemma 1, because for all indices i,1≤i≤n, there exists a row vector which has Hamming weight of three and one non-zero element in the *i*-th position in $H^{\prime }$, the proposed BLRCs have all-symbol locality r=2. □

In addition, the proposed BLRCs have uneven availabilities while achieving the locality r=2 as follows.

**Theorem 2** (Availability of the proposed BLRCs)**.** *An $\left(n,k\right)$ BLRC C with Construction 1 has all-symbol availability profile represented as*

(9) $$t=[\underset{k}{\underset{︸}{k-1,\cdots ,k-1}},\;\underset{k\left(k-3\right)}{\underset{︸}{2,\cdots ,2}},\;\underset{k}{\underset{︸}{1,\cdots ,1}}]$$

*for local repair with all-symbol locality r=2.*

**Proof.**  To prove this, $H^{\prime }$ in Equation (8) is used again. The first *k* column vectors and the next *k* column vectors of $Q_{k,1}^{T}$ have Hamming weights of two and one, respectively, whereas the remaining column vectors have a Hamming weight of zero. In $Q_{k,l}^{T}+Q_{k,l+1}^{T},\;1\leq l\leq k-3$, the first *k* column vectors have Hamming weight one and the column vectors from the (lk+1)-th position to the (lk+2k)-th position also have Hamming weight one, whereas the remaining column vectors have a Hamming weight of zero. Therefore, the first *k* column vectors, the next $k\left(k-3\right)$ column vectors, and the last *k* column vectors have Hamming weights of k−1, two, and one, respectively.

Now, we must show that all local repair groups which repair the same error symbol are disjoint. For every index i, 1≤i≤k, there are k−1 row vectors of which each has one in the *i*-th index and the other *r* ones in disjoint positions, except for the *i*-th position in $H^{\prime }$. In addition, for every index i, k+1≤i≤k(k−2), there are two row vectors of which each has one in the *i*-th position and other *r* ones in disjoint positions, except for the *i*-th position in $H^{\prime }$. Lastly, for every index i, k(k−2)+1≤i≤k(k−1), there is a row vector which has one in the *i*-th position in $H^{\prime }$. Therefore, according to Lemma 2, the proposed BLRCs have the uneven all-symbol availability profile expressed in Equation (9). □

Note that the proposed BLRCs C have constant information symbol availability k−1 but the uneven all-symbol availabilities for local repair.

**Figure 2:**
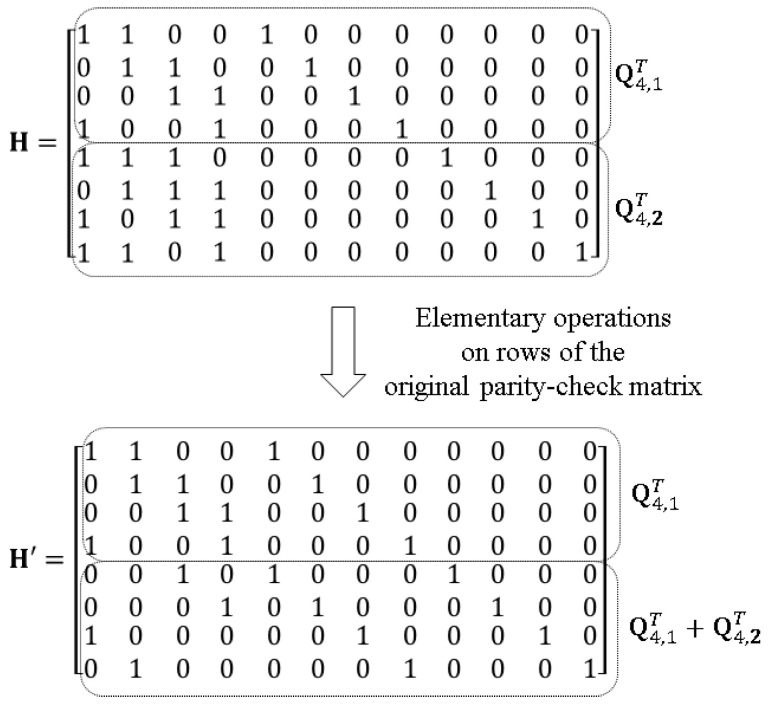
An example of a parity-check matrix and its modified form in the proposed $\left(12,4,2,[3,3,3,3,2,2,2,2,1,1,1,1],6\right)$ BLRCs.

**Example** **2.** *The proposed $\left(n=12,k=4,r=2\right)$ BLRC C constructed in Example 1 has a parity-check matrix H and the modified form $H^{\prime }$ as shown in Figure 2. It is verified in $H^{\prime }$ that C has all-symbol locality r=2. In addition, C has the all-symbol availability profile $t=\left[3\;3\;3\;3\;2\;2\;2\;2\;1\;1\;1\;1\right]$.*

### 3.3. Optimality of the Proposed BLRCs

In this subsection, the optimality of proposed BLRCs is evaluated in terms of the bound in Equation (2) for given code length and minimum Hamming distance, while achieving high performance with regard to locality and uneven availabilities. Initially, in order to determine the minimum Hamming distance of the proposed BLRCs, the following lemma is used.

**Lemma 3** (Minimum Hamming distance by parity-check matrix)**.** *The minimum Hamming distance of a code is equal to the smallest number of column vectors of its parity check matrix, which form a linearly dependent set.*

For a given information length *k*, the minimum Hamming distance of the proposed BLRCs is determined as shown below.

**Theorem 3** (Minimum Hamming distance of the proposed BLRCs)**.** *An $\left(n,k\right)$ BLRC C with Construction 1 has a minimum Hamming distance of d=2k−2 for a given k.*

**Proof.**  It is verified in the parity check matrix H of C in Equation (6) and (7) that for a given *k*, two column vectors selected properly from the first *k* column vectors have a minimum Hamming distance of $2\left(k-2\right)$ and thus can be represented as $2\left(k-2\right)$ column vectors selected properly from the last n−k column vectors. Thus, these selected 2k−2 column vectors form a linearly dependent set, of which the cardinality is smallest in the linearly dependent sets of the column vectors in H. Therefore, the minimum Hamming distance *d* of C is 2k−2. □

**Example** **3.** *The proposed $\left(n=12,k=4\right)$ BLRC C constructed in Example 1 has the parity-check matrix H shown in Figure 2. It is easily verified in H that C has a minimum Hamming distance of d=6.*

The proposed BLRCs achieve optimality in terms of the bound in Equation (2) for k=4 as follows.

**Theorem 4** (Optimality of the proposed BLRCs)**.** *For k=4, a $\left(k\left(k-1\right),\;k,\;2,\;1,\;2\left(k-1\right)\right)$ BLRC C with Construction 1 achieves optimality in terms of the bound in Equation (2).*

**Proof.**  In Reference [17], $\left(2^{m}-4,\;m,\;2,\;1,\;2^{m-1}-2\right)$ BLRCs for m≥4 was proved to have the bound in Equation (2). The information length *k* in C corresponds to *m* and for k=4, $\left(12,4,2,\left[3,3,3,3,2,2,2,2,1,1,1,1\right],6\right)$ BLRCs can be constructed by Construction 1. Therefore, $\left(12,4,2,\left[3,3,3,3,2,2,2,2,1,1,1,1\right],6\right)$ BLRCs with Construction 1 achieve the bound in Equation (2). □

The BLRCs in two earlier studies [16,17] also achieve the bound in Equation (2) and we compare those codes with our proposed BLRCs in Table 1. S-BLRC represents the simplex code in Reference [16], A-BLRC represents the binary LRC constructed from anti-codes in Reference [17], and P-BLRC represents our proposed BLRC. Remind that S-BLRC meets the bound in Equation (2) for all *k* while A-BLRC does so for only 4≤k≤6. Though P-BLRC meets the bound for only k=4, P-BLRC has a higher rate and more abundant choice of *n* than the others and the number of symbols with availability two or more in P-BLRC is larger than that of the others.

**Remark** **1.** *Most of the local repair groups in the proposed BLRCs have one information symbol and two parity symbols. This means that the proposed BLRCs can access no other information symbols, but only the parity symbols for the local repair of each information. This property is desirable for hot data because the network traffic for temporal repair of an information symbol can be distributed to parity symbols so that congestion of traffic around hot information data is avoided.*

**Remark** **2.** *Despite the fact that the condition that every local repair group has only one local parity symbol is not satisfied, the proposed BLRCs with k=4 achieve the bound in Equation (3). However, given that the local repair groups in the proposed LRCs have not only one parity symbol but also two parity symbols, an evaluation of the proposed BLRCs with regard to the optimality of the minimum Hamming distance requires stricter conditions compared to those in Equation (3). As further research, a bound tighter than that in Equation (3) should be derived to evaluate optimality for the minimum Hamming distance of the proposed BLRCs.*

## 4. Conclusions

In this paper, a family of BLRCs is proposed, which have all-symbol locality two and a high information symbol availability and low parity symbol availabilities, that is, good uneven all-symbol availabilities for the local repair. The proposed BLRCs with k=4 achieve optimality for the information length while maintaining high performance on locality, availability, and the minimum Hamming distance.

## Figures and Tables

**Table 1:** Parameter comparison of BLRCs in References [16,17], and this paper.

Parameter	S-BLRC [16]	A-BLRC [17]	P-BLRC
Code length (*n*)	$2^{k}-1$	$2^{k}-\frac{1}{2}k^{2}+\frac{3}{2}k-2$	$k^{2}-k$
Code rate	$\frac{k}{2^{k}-1}$	$\frac{2k}{2^{k+1}-k^{2}+3k-2}$	$\frac{1}{k-1}$
Locality	2	2	2
Number of symbols with availability 2 or more	$n-2^{k-1}$	n−2k+1	n−k
Minimum distance	$2^{k-1}$	$2^{k-1}-\left\lfloor \frac{{\left(k-1\right)}^{2}}{4}\right\rfloor$	2k−2
